# Supplementary material for: Evaluation of cell segmentation methods without reference segmentations
Source: Mol Biol Cell. 2023 May 5;34(6):ar50. doi: 10.1091/mbc.E22-08-0364 (PMC10208095; doi:10.1091/mbc.E22-08-0364)
Supplement: Supplementary file 2 [file mbc-34-ar50-s002.pdf]

Evaluation of cell segmentation methods without reference segmentations

Haoran Chen and Robert Murphy

Corresponding author(s): Robert Murphy, Carnegie Mellon University

**Review Timeline:**		
	Submission Date:	2022-08-24
	Editorial Decision:	2022-10-11
	Revision Received:	2022-11-27
	Accepted:	2022-11-29

Editor-in-Chief: Matthew Welch

Transaction Report:

(Note: With the exception of the correction of typographical or spelling errors that could be a source of ambiguity, letters and reports are not edited. The original formatting of letters and referee reports may not be reflected in this compilation.)

RE: Manuscript #E22-08-0364
TITLE: Evaluation of cell segmentation methods without reference segmentations

Dear Dr. Murphy:

Your manuscript, entitled " Evaluation of cell segmentation methods without reference segmentations" has been seen by two referees whose verbatim comments are enclosed. Both referees felt that your findings are of interest to our MBoC readership. It is important to consider the suggestion by Reviewer #1 to compare your algorithm with available ground truth data or other benchmarks and Reviewer #2's suggestion to expand the discussion on limitations. These additions will make the algorithm more accessible to biologist as end users. We look forward to receiving your revised manuscript and a letter indicating your response to the referees in the near future.

Sincerely,

Diane Lidke
Monitoring Editor
Molecular Biology of the Cell

Dear Dr. Murphy,

The review of your manuscript, referenced above, is now complete. The Monitoring Editor has decided that your manuscript is not acceptable for publication at this time, but may be deemed acceptable after specific revisions are made, as described in the Monitoring Editor's decision letter above and the reviewer comments below.

A reminder: Please do not contact the Monitoring Editor directly regarding your manuscript. If you have any questions regarding the review process or the decision, please contact the MBoC Editorial Office (mboc@ascb.org).

When submitting your revision include a rebuttal letter that details, point-by-point, how the Monitoring Editor's and reviewers' comments have been addressed. (The file type for this letter must be "rebuttal letter"; do not include your response to the Monitoring Editor and reviewers in a "cover letter.") Please bear in mind that your rebuttal letter will be published with your paper if it is accepted, unless you haveopted out of publishing the review history.

Authors are allowed 180 days to submit a revision. If this time period is inadequate, please contact us at mboc@ascb.org.

Revised manuscripts are assigned to the original Monitoring Editor whenever possible. However, special circumstances may preclude this. Also, revised manuscripts are often sent out for re-review, usually to the original reviewers when possible. The Monitoring Editor may solicit additional reviews if it is deemed necessary to render a completely informed decision.

In preparing your revised manuscript, please follow the instruction in the Information for Authors (www.molbiolcell.org/info-for-authors). In particular, to prepare for the possible acceptance of your revised manuscript, submit final, publication-quality figures with your revision as described.

To submit the rebuttal letter, revised manuscript, and figures, use this link: Link Not Available

Please contact us with any questions at mboc@ascb.org.

Thank you for submitting your manuscript to Molecular Biology of the Cell. We look forward to receiving your revised paper.

Sincerely,

Eric Baker
Journal Production Manager
MBoC Editorial Office
mbc@ascb.org

Reviewer #1 (Remarks to the Author):

Chen and Murphy present a method for benchmarking of segmentation algorithms without ground truth data. They suggest several new and creative metrics for label-free benchmarking and share documented code to run their approach. However, the authors do not compare to standard benchmarks for cellular segmentation, which means a biologist cannot compare these metrics to metrics presented in other papers to decide if they are adequate for evaluating quality.
There are two options for the authors. The first is to compute their metrics on a large dataset with ground truth. There is substantial ground truth data available from the DeepCell paper as well as a recent LiveCell paper. Given the DeepCell/TissueNet dataset is similar to the dataset in the papers, I think it would greatly strengthen the paper to show that their PC1 of the metrics correlate with benchmarks like the F1 score across models.
Alternatively, the authors could label a few images from their training set and compute the F1 score of the models and see if it correlates with their benchmarks.
Either of these approaches would scientifically validate the findings of the paper and thus increase the adoption of the tool in the segmentation community.

Minor points:
* The DeepCell package has multiple network architectures available, can the authors specify which one was used? Also there is a new DeepCell model released with the Greenwald, Miller et al paper that was trained on more data that may perform better.
* Cellpose now has a Tissuenet model that might be more appropriate for comparison with the data in the current paper.
* import->importance

Reviewer #2 (Remarks to the Author):

The manuscript `Evaluation of cell segmentation methods without reference segmentations´by Haoran Chen and Robert F. Murphy address the problem of assessing cell segmentation methods in the absence of manually defined cell masks. The authors propose to use a combination of metrices, that are combined in a principal component analysis and ranked for the comparison of different deep learning frameworks for cell segmentation.

This approach is of general high relevance for the analysis of multi-plex images from different tissues and imaging methods. The manuscript is well structured and the data are presented in a clear manner. Code and a tool are made publicly available.

The strength of this work lies in the assessment of segmentation accuracy and robustness using 14 metrics defined based on prior knowledge of cell and image properties in combination with PCA. This approach allows the comparison of several widely used cell/nucleus segmentation methods. Interestingly, this shows that human expert annotators differ quite substantially as compared to e.g. DL algorithms, such as Cellpose and DeepCell.

One weakness of the approach is that the segmentation performance on unusual, difficult to segment cell and nuclear shapes might not be adequately reflected in the assessment. Novel methods addressing these, will most likely also in the future rely on manual annotations of cell or nucleus masks. This and other potential limitations as confounding factors, such as variation in resolution among imaging methods and cell distribution and shapes among tissue types vs cultured cells should be discussed.

Computational Biology Department | Carnegie Mellon University School of Computer Science

November 27, 2022

Dr. Diane Lidke
Monitoring Editor
Molecular Biology of the Cell

Dear Diane,

We thank you for the opportunity to revise the manuscript entitled "Evaluation of cell segmentation methods without reference segmentations" for publication in MBoC. We also thank the reviewers for the helpful comments. We have carefully reviewed the comments and have revised the manuscript accordingly.

Reviewer #1 (Remarks to the Author):

Chen and Murphy present a method for benchmarking of segmentation algorithms without ground truth data. They suggest several new and creative metrics for label-free benchmarking and share documented code to run their approach. However, the authors do not compare to standard benchmarks for cellular segmentation, which means a biologist cannot compare these metrics to metrics presented in other papers to decide if they are adequate for evaluating quality.
There are two options for the authors. The first is to compute their metrics on a large dataset with ground truth. There is substantial ground truth data available from the DeepCell paper as well as a recent LiveCell paper. Given the DeepCell/TissueNet dataset is similar to the dataset in the papers, I think it would greatly strengthen the paper to show that their PC1 of the metrics correlate with benchmarks like the F1 score across models.
Alternatively, the authors could label a few images from their training set and compute the F1 score of the models and see if it correlates with their benchmarks.
Either of these approaches would scientifically validate the findings of the paper and thus increase the adoption of the tool in the segmentation community.

Comparing our quality scores to traditional measures that require a human expert is a great suggestion. We have done this and feel that it significantly improves our manuscript.

The TissueNet dataset does not provide original multi-channel images but only two channels as segmentation inputs. Hence, it is not suitable for evaluation using our pipeline since our metrics in large part rely upon having multiple channels to measure homogeneity at the cell level. Alternatively, as the reviewer suggested, we calculated three benchmarks (including F1 score) for the human annotated images we originally evaluated with our metrics. We compared these benchmarks to our quality score for each segmentation method/expert annotation on each image (Figure 6). Our quality score highly correlates with all three benchmarks (0.83, 0.86 and 0.72 respectively).

Figure 6, a section in Methods, and a section in Results were added to discuss this.

Minor points:
* The DeepCell package has multiple network architectures available, can the authors specify which one was used? Also there is a new DeepCell model released with the Greenwald, Miller et al paper that was trained on more data that may perform better.

We clarified that we used the PanopticNets architecture, which was derived from the Lin et al, 2017 paper, in the DeepCell package.
We also added evaluation of the latest version of DeepCell (v0.12.3) as well as the latest version of Cellpose (v2.1.0) on all images. We updated the PCA model along with all figures, supplementary figures, tables and descriptions to incorporate the new results. We also moved results for the older versions of DeepCell and CellPose to a new supplementary figure (Supplementary Figure 3).

* Cellpose now has a Tissuenet model that might be more appropriate for comparison with the data in the current paper.

We tried to apply Cellpose with its TissueNet model, but it segmented none to few cells for multiple images regardless of the choice of cell diameter parameters. Hence, we decided not to use it.

* import->importance

fixed.

Reviewer #2 (Remarks to the Author):

The manuscript `Evaluation of cell segmentation methods without reference segmentations´by Haoran Chen and Robert F. Murphy address the problem of assessing cell segmentation methods in the absence of manually defined cell masks. The authors propose to use a combination of metrices, that are combined in a principal component analysis and ranked for the comparison of different deep learning frameworks for cell segmentation.

This approach is of general high relevance for the analysis of multi-plex images from different tissues and imaging methods. The manuscript is well structured and the data are presented in a clear manner. Code and a tool are made publicly available.

The strength of this work lies in the assessment of segmentation accuracy and robustness using 14 metrics defined based on prior knowledge of cell and image properties in combination with PCA. This approach allows the comparison of several widely used cell/nucleus segmentation methods. Interestingly, this shows that human expert annotators differ quite substantially as compared to e.g. DL algorithms, such as Cellpose and DeepCell.

One weakness of the approach is that the segmentation performance on unusual, difficult to segment cell and nuclear shapes might not be adequately reflected in the assessment. Novel methods addressing these, will most likely also in the future rely on manual annotations of cell or nucleus masks. This and other potential limitations as confounding factors, such as variation in resolution among imaging methods and cell distribution and shapes among tissue types vs cultured cells should be discussed.

We have addressed these points in the Discussion.

We hope that with these revisions the manuscript will now be acceptable.

With best regards,

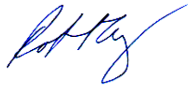

Robert F. Murphy, Ph.D.
Ray and Stephanie Lane Professor of Computational Biology Emeritus
IEEE Fellow, AIMBE Fellow

RE: Manuscript #E22-08-0364R
TITLE: "Evaluation of cell segmentation methods without reference segmentations"

Dear Dr. Murphy:

Thank you for revising your manuscript in response to the referees' recommendations. I am pleased to accept your manuscript for publication in MBoC. Congratulations!

Sincerely,
Diane Lidke
Monitoring Editor
Molecular Biology of the Cell

Dear Dr. Murphy:

Congratulations on the acceptance of your manuscript.

A PDF of your manuscript will be published on MBoC in Press, an early release version of the journal, within 10 days. The date your manuscript appears at www.molbiolcell.org/toc/mboc/0/0 is the official publication date. Your manuscript will also be scheduled for publication in the next available issue of MBoC.

Within approximately four weeks you will receive a PDF page proof of your article.

Would you like to see an image related to your accepted manuscript on the cover of MBoC? Please contact the MBoC Editorial Office at mboc@ascb.org to learn how to submit an image.

Authors of Articles and Brief Communications are encouraged to create a short video abstract to accompany their article when it is published. These video abstracts, known as Science Sketches, are up to 2 minutes long and will be published on YouTube and then embedded in the article abstract. Science Sketch Editors on the MBoC Editorial Board will provide guidance as you prepare your video. Information about how to prepare and submit a video abstract is available at www.molbiolcell.org/science-sketches. Please contact mboc@ascb.org if you are interested in creating a Science Sketch.

We are pleased that you chose to publish your work in MBoC.

Sincerely,

Eric Baker
Journal Production Manager
MBoC Editorial Office
mbc@ascb.org
